# Unique Challenges of Hebrew Translation and Cross-Cultural Adaptation of LIMB-Q Kids for Children with Lower Limb Differences

**DOI:** 10.3390/children12101318

**Published:** 2025-10-01

**Authors:** Sharon Eylon, Michal Lieberman, Gilad Brandes, Patrice L. Weiss, Vladimir Goldman, Anthony P. Cooper, Harpreet Chhina

**Affiliations:** 1 ALYN Hospital Pediatric & Adolescent Rehabilitation Center, 84 Shmariyahu Levin St., Jerusalem 91090, Israel; michall@alyn.org; 2 The Helmsley Pediatric & Adolescent Rehabilitation Research Center, Jerusalem 91090, Israel; tamarw@alyn.org; 3 Department of Communication Disorders, Jerusalem Multidisciplinary College, Jerusalem 91010, Israel; giladbr@edu.jmc.ac.il; 4 Department of Occupational Therapy, University of Haifa, Haifa 31905, Israel; 5 Department of Orthopedic Surgery, Hadassah Medical Center, School of Medicine, Hebrew University of Jerusalem, Jerusalem 91120, Israel; goldmanv@hadassah.org.il; 6 Department of Orthopaedics, BC Children’s Hospital, Vancouver, BC V6H 3V4, Canada; anthony.cooper@cw.bc.ca (A.P.C.); hchhina@cw.bc.ca (H.C.); 7 Department of Orthopaedics, Faculty of Medicine, University of British Columbia, Vancouver, BC V5Z 1M9, Canada

**Keywords:** patient-reported outcome measure, clinical outcomes and patient experience, quality of life, lower limb discrepancy

## Abstract

Background/Objectives. Patient-Reported Outcome Measures (PROMs) capture patients’ perspectives about their health status, quality of life, and medical care outcomes. LIMB-Q Kids is a validated PROM designed to assess health-related quality of life (HRQL) in children with lower limb differences. It evaluates physical, social, and psychological function; symptoms related to the leg, hip, knee, foot and ankle; leg-related distress, appearance, and school-related concerns. It has now been translated and culturally adapted from English to other languages. The aim of this study was to translate and culturally adapt LIMB-Q Kids to Hebrew. Methods. Following international guidelines, two independent forward translations from English to Hebrew were produced and reconciled into a single version. A backward translation was then compared with the original to identify discrepancies. This draft underwent cognitive debriefing interviews (CDIs) with 11 children (ages 8–15) having lower limb differences to assess comprehension and cultural relevance. Results. CDIs revealed general issues including lengthy or unclear text, high-level language, cultural unfamiliarity and duplication of descriptors. Specific to Hebrew, issues included gender inflections and the need for vowel diacritics to support younger, less proficient readers. Revisions to 14 items were made. Conclusions. A translation and cultural adaption (TCA) process led to a linguistically validated and culturally adapted Hebrew version of LIMB-Q Kids. It can now be used for the clinical follow-up of children with lower limb differences including pre- and post-operatively, and as an aid to decision-making for surgery.

## 1. Introduction

Patient-Reported Outcome Measures (PROMs) are standardized tools that collect patients’ own assessments of their health, symptoms, and quality of life, without interpretation by clinicians or others [1]. They offer valuable insight into patient experiences, complementing clinical outcomes and supporting more patient-centered care [2]. PROMs, developed through rigorous validation, are used to evaluate symptom severity, functional ability, and care satisfaction. PROMs may be generic (i.e., applicable to a wide range of conditions and populations) or condition/disease specific. Their growing role in clinical trials and health policy helps inform treatment decisions and reimbursement strategies.

Since PROMs are language-based instruments, their use across different countries necessitates a rigorous methodological translation process. A successful translation must maintain accuracy and fidelity to the original version to preserve validity and reliability, while simultaneously ensuring natural readability and maximum clarity for speakers of the target language. This dual requirement often necessitates culturally sensitive adaptations tailored to specific linguistic and cultural contexts. Patient literacy and comprehension levels present additional complexity, as variation in these factors can significantly impact data accuracy and reliability [3]. Particular attention must be paid to ensuring appropriate language complexity (e.g., vocabulary and syntax) for diverse patient populations, which is especially critical when working with pediatric populations who may have limited reading and comprehension abilities. Furthermore, PROMs must authentically reflect cultural variations in how health and well-being are conceptualized, experienced, and valued [2]. For instance, children from culturally conservative backgrounds that emphasize modesty may interpret and respond to appearance-related questions differently than those from less traditional environments, highlighting the need for cultural sensitivity in both translation and adaptation processes.

Lower limb differences arise from various causes, such as congenital limb deficiencies, trauma, growth disturbances, or tumors, that are expressed as leg length discrepancy and/or angular differences [4]. These often lead to physical limitations, pain, and reduced participation in activities which can affect children’s development and adjustment. Factors influencing participation include social support, functional abilities, and personal traits like age and gender. Treatment options range from conservative approaches (e.g., insoles, prostheses, orthotics) to surgical interventions, including growth arrest, lengthening, reconstructive surgery, or amputation. Severe differences often require challenging decisions between reconstruction and amputation, with limited evidence to guide these choices. The lack of studies comparing treatment options complicates informed decision-making. Planning and evaluating treatment require objective measures such as radiographs as well as the patient’s perspective to ensure patient-centered care. Chhina et al. [5] identified this gap through a systematic review, leading to the creation of the LIMB-Q Kids PROM, a validated tool to assess health-related quality of life (HRQL) in children with lower limb differences [5,6]. The concept elicitation and content validation studies resulted in a version of LIMB-Q Kids that comprised 11 independently functioning scales measuring physical, social, and psychological functions; symptoms related to the leg, hip, knee, ankle and foot; leg-related distress, appearance, school-related concerns and scars resulting from surgeries [7,8].

This version of LIMB-Q Kids was internationally field-tested across 16 sites in seven countries, including English-speaking nations (Australia, Canada, the USA, and the UK) as well as non-English-speaking countries where it was translated and culturally adapted accordingly (Danish [7], Finnish, German [8], Hindi [9], and Portuguese). It is important to continue to diversify these assessments to be able to include patients of all backgrounds and languages. Hence, an Israeli team collaborated with researchers at the University of British Columbia to translate and culturally adapt LIMB-Q Kids to Hebrew, employing specific adjustments to accommodate the unique characteristics of this language.

## 2. Methods

### 2.1. Translation and Cultural Adaptation (TCA)

**Figure 1:**
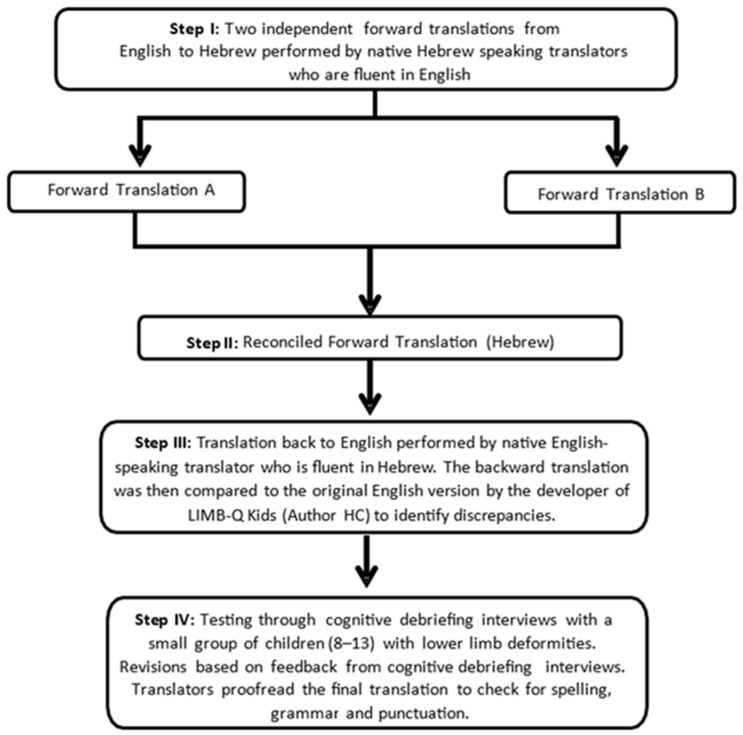
Flow diagram of the translation and cultural adaptation protocol for the LIMB-Q Kids as recommended by the test developers.

LIMB-Q Kids was translated and culturally adapted into Hebrew, following the translation and cultural adaptation (TCA) flowchart shown in Figure 1. In accordance with the ISPOR guidelines [10], the TCA process began with two independent forward translations from English to Hebrew performed by professional translators who are fluent in English and native speakers of Hebrew. These translations, referred to as Forward Translation A and Forward Translation B, were then combined into a single reconciled version through collaboration and review. This version was subsequently translated back into English in a process called backward translation. It was carried out by a translator who is a native English speaker. The backward translation was compared to the original English version by the developer of LIMB-Q Kids (Author HC) to identify discrepancies. Revisions were then made by the core research team (Authors SE, ML, PLW, HC) to ensure accuracy and alignment with the source text. Under the guidance of a UBC researcher (HC) who has extensive experience with multiple language translations of the LIMB-Q Kids, this version was used for cognitive debriefing interviews (CDIs, described below) where the translation was tested with a small group of children to assess the clarity and comprehensibility of the translation. Feedback from these interviews was used to make further revisions. Finally, the researchers proofread the refined translation, checking for errors in spelling, grammar, and punctuation.

### 2.2. Participants

The sample included children aged 8–15 years, native Hebrew speakers, with a confirmed diagnosis of any lower limb difference including children with various types of lower limb differences (e.g., congenital limb length discrepancies or acquired limb length discrepancies due to trauma/oncology, amputations, or any other types of differences with LLD). Children with lower limb differences and other medical conditions such as cognitive or developmental delay affecting their ability to read and write independently were excluded from participation in this study. Isolated hip, knee and foot pathologies were excluded. The child’s age at the time of CDI data collection, sex, type of discrepancy/impairment and treatment were recorded.

Ethical approval for this study was received from the hospital’s Helsinki Committee for Ethical Research of humans (#069-22). Informed consent from patients and parents was obtained by a designated member of the research team.

### 2.3. Cognitive Debriefing Interviews

In accordance with the ISPOR guidelines for CDIs [10], the participant was given the LIMB-Q Kids questionnaire and shown a figure of the lower limbs with labeled joints and segments (Supplementary Materials). The child was then requested to read each instruction, response option and item out loud. Following each word or phrase, the participant was asked to describe the read content in their own words and confirm its meaning. In cases where the child was unable to comprehend or describe the meaning, the research assistant gave the child an opportunity to elaborate on their difficulty. The participating children were encouraged to provide suggestions for rewording or rephrasing any difficult items or instructions.

## 3. Results

The forward and back translations proceeded as indicated in Figure 1. This was accompanied by consultation with the developers of LIMB-Q Kids who provided advice and feedback as the data were collected.

In total, 14 children were recruited for the CDIs. One child dropped out before completing the process, and two children, aged 15.5 and 11 years, faced unexpected difficulties with reading. Ultimately, a group of 11 children (four girls, seven boys), aged 8–13 years (mean ± SD = 10.6 ± 1.7), were included. (Table 1). Note that ISPOR guidelines recommend a minimum of 5–8 cognitive debriefing interviews [10].

During the CDIs, a number of key issues related to the presentation of the text or specific Hebrew language requirements arose (Table 2). Specific examples are provided to illustrate each issue.

I. Text too long or lacks clarity. For example, the Hebrew text “If you wear a special shoe, an insole, an orthosis, or have a prosthetic leg, we ask that you answer the questions while considering the time you use this device…” was long and convoluted and the children found it difficult to follow. It was changed to: “If you use an assistive device such as a shoe lift, insole, brace, or prosthetic leg, answer the questions while considering the time during which you use this device”. In other items, the text was unclear. For example, in the instruction “These questions pertain to the leg for which you are seeing a doctor or nurse”, the children did not find the reference to a visit to a doctor or nurse to help them identify the affected limb. We changed this to “This question refers to the leg for which you are seeing a doctor.”

II. Level of language too high. Children had difficulty understanding some Hebrew words or phrases such as, “How well do your legs match each other?” We changed this to “Are your legs similar to each other (do they look the same)?” In some cases, more sophisticated prepositions were changed to simpler forms (e.g., *al* ‘about’ instead of *legabey* ‘regarding’). Here and throughout the paper, transliterations of Hebrew words are given in italics.

III. Lack of cultural familiarity. Certain terms, such as the one used in Hebrew to refer to sitting cross-legged, “Eastern sitting”, was not understood by some of the children (e.g., ultra-Orthodox). Here we used an additional, more literal description: “sitting cross-legged”.

IV. Duplication of descriptors. A few items proved difficult to translate precisely due to English expressions that use synonyms which correspond to the same Hebrew word. For example, the adjectives ‘swollen’ and ‘puffy’ both translate most naturally into Hebrew as *nafuax*. Initially, an attempt was made to preserve the original phrasing by using a near-synonym, *tafuax*, even though the phrase *nafuax ve-tafuax* does not sound as natural in Hebrew as the English ‘swollen and puffy’. A similar case was the phrase ‘rest and relax’, which was initially translated literally using the synonyms *menuxa* and *regi’a*. However, following participants’ comments, these expressions were revised to more natural Hebrew by omitting the second descriptor—resulting in *nafuax* (‘swollen’) and *menuxa* (‘rest’).

V. Use of male and female endings. Unlike English, all Hebrew verbs, adjectives, animate nouns, pronouns, and other word classes inflect to mark gender (e.g., *lovesh =* wears.MASCULINE versus *loveshet* = wears.FEMININE). As is often done in Hebrew materials, the original translation used a dual-gender format, with the feminine suffix added after a slash (e.g., *lovesh/et*). This issue was addressed by one participant, who remarked that this format confused him.

VI. Use of vowel diacritics. The Hebrew script is an *abjad*, which represents consonants consistently while leaving vowel information largely absent—requiring readers to infer it from context. For example, the sequence SFR (ספר), which contains no vowel indication, can be read as different words, including *sefer* ‘book’ and *safar* ‘he counted’. Typically, this does not pose a difficulty for proficient readers, but may be challenging, or even make decoding impossible, for novice or young readers. Therefore, novice readers typically use the pointed version of the Hebrew script, which incorporates specialized diacritic-like signs called *nikud.* These signs, which appear mostly underneath the letters, provide full vowel information (as in סֶפֶר *sefer* ‘book’). In the present study, participants were shown an unpointed version of the translated LIMB-Q Kids. Four participants remarked that they found this difficult and would have preferred a pointed version which is in line with research on the development of reading proficiency in Hebrew [11].

The final Hebrew version of LIMB-Q Kids includes 14 text item changes in categories I-IV (3 instructions and 11 items) as shown in Table 2, last row. Categories V and VI relate to the entire PROM text. Translation into Hebrew included the final 89 items of LIMB-Q Kids spread across 9 independently functioning scales based on the field test study [6], resulting in four versions of LIMB-Q Kids Hebrew: A pointed (P) and unpointed (UP) version for each gender (M/F) (LIMB-Q Kids H-M-P; LIMB-Q Kids H-F-P; LIMB-Q Kids H-M-UP; and LIMB-Q Kids H-F-UP). These are provided in Supplement SII.

## 4. Discussion

Given the significant impact of lower limb differences on physical function, emotional well-being, and quality of life, the use of a PROM is essential to capture the patient’s perspective and provide a comprehensive and functionally meaningful evaluation of treatment outcomes. PROMs allow children to self-report when historically their perspectives have been underrepresented or overlooked in clinical decision-making and research [12]. This goal led to the development of LIMB-Q Kids [5,6] and adoption in other languages [7,8,9].

One may be tempted to simply use the English version of the LIMB-Q Kids, assuming that children aged 8 years and older would be able to provide accurate responses. In our clinical experience, this would seriously impede the data reported by the child. We therefore chose to invest in the full translation process. Although it may be tempting to rely on the English version of PROMs, we firmly believe that administering them in a language other than the child’s native language undermines both the quality and the accuracy of the data reported. Appropriate translation and cultural adaptation of PROMs ensures conceptual equivalence, improves data accuracy and reliability, reduces response bias, enhances inclusivity and equity and supports cross cultural comparisons [10].

Although the process of linguistic and cultural adaptation to different languages is similar, there are considerable differences in the final product which reflects factors related to specific issues (e.g., use of vowel markings, gender and age of reaching reading proficiency). Hence the process described in this paper, and the unique issues it raises, are expected to support the translation of LIMB-Q Kids and other tools to diverse languages.

For example, in contrast to preparing masculine and feminine versions of the Hebrew LIMB-Q Kids, the Hindi LIMB-Q Kids selected to make majority of the items gender-neutral [9]. Though there were some items that could not be made gender neutral and provided options for both male and female participants. This solution was not applicable in Hebrew since it resulted in a very awkward syntax. Future translators of other gender specific languages such as Arabic will benefit from comparing the Hebrew and Hindi ways of accommodating gender in their versions of the LIMB-Q Kids.

The current study describes the process of translating and culturally adapting the LIMB-Q Kids to Hebrew and emphasized the unique challenges related to this language. On the one hand, the process revealed many similarities to issues encountered in other languages such as German and Danish. For example, Jønsson et al. [7], p. 6 describe the problem of Lack of cultural familiarity: “’sitting cross-legged’ which is often described as a literal translation of the expression ‘Tailor sitting position’ in Danish. This expression was combined with the literal translation of ‘sitting cross-legged’ in a bracket. Some of the younger children were confused as they did not know this expression. It was therefore decided to add the expression in brackets instead of the literal translation to ensure the understandability of the item.” In the German translation, Vogt et al. [8], p. 4 also found an issue with a Duplication of descriptors (e.g., ‘swollen and puffy”) and made a similar decision to retain only one German word instead of the two English ones.

In contrast, distinct challenges arose in this study during the TCA to Hebrew, a Semitic language whose grammatical structure and writing system diverge markedly from those of most Indo-European languages. The most relevant issues in this context were gender marking and the lack of orthographic vowel representation.

As described above, gender is a pervasive aspect of Hebrew grammar. There are two common strategies for the writer using only one gender form throughout the text or adding the feminine suffix after a slash or a period (as exemplified above). While the former strategy necessarily excludes readers of one gender (typically females), the latter can seem cumbersome and may confuse non-proficient readers. For this reason, two single-gender versions of the Hebrew LIMB-Q Kids were created, one for girls and one for boys. It is surprising that the issue of gender had not been highlighted earlier, given its clear relevance in languages such as German [8] and Danish [7]. Gender-based issues did arise during translation of LIMB-Q Kids into Hindi [9]. However, the authors of the Hindi translation addressed this issue by making several items gender neutral and tested them during the CDIs; the children were able to understand these gender-neutral items easily. This solution, however, would not be appropriate for Hebrew, where gender distinctions are deeply embedded in grammar and cannot usually be neutralized without creating awkward or cumbersome phrasing. This approach is also in line with the recommendations of the Academy of the Hebrew Language [13].

A second issue concerned the distinction between pointed and unpointed Hebrew scripts. Early reading materials use the pointed version, which represents vowels with diacritics, but these markings are largely absent from texts for adolescents and adults. Consequently, young readers rely on diacritics for decoding, while proficient readers use morphology, vocabulary, and context—and for them, diacritics can increase cognitive load [14,15,16,17]. In line with this, younger participants in the present study preferred pointed text, whereas older readers found it unnecessary or distracting. To address both needs, pointed and unpointed versions of the Hebrew LIMB-Q Kids were created, ensuring suitability for novice readers [18].

## 5. Conclusions

LIMB-Q Kids is a rigorously developed PROM that captures the multifaceted experiences of children with lower limb differences, facilitating better assessment and targeted interventions to enhance their HRQL. Given the special concerns of gender and of vowel markings in the Hebrew language, four versions of Hebrew Limb-Q Kids are now available for use. The process described in this study, and the unique issues raised, may support the translation of LIMB-Q Kids and other tools into diverse languages. The growing role of PROMs in clinical trials and health policy is essential for treatment decisions and reimbursement strategies.

## Figures and Tables

**Table 1:** Demographic and medical data.

Child	Age (Years)	Gender	Diagnosis	On-Going Treatment at the Time of CDI	Past Treatment
1	11	F	Achondroplasia	Lengthening with external fixator + Epiphysiodesis	None
2	13	M	Trauma	Epiphysiodesis	Lengthening with magnetic nail
3	9	M	Lower limb Congenital discrepancies	Lengthening with external fixator	None
4	11	M	Lower limb Congenital discrepancies	Lengthening with magnetic nail	Lengthening with external fixator; Epiphysiodesis
5	12	M	Genetic unilateral Leg Length discrepancy or deformity	Lengthening with magnetic nail	Lengthening with external fixator; Epiphysiodesis
6	12	M	Genetic unilateral Leg Length discrepancy or deformity	Lengthening with magnetic nail	Lengthening with external fixator
7	12	M	Achondroplasia	Lengthening with external fixator	None
8	11	F	Trauma	Lengthening with magnetic nail	Epiphysiodesis
9	8	M	Congenital discrepancies of lower limb	Lengthening with magnetic nail + Epiphysiodesis	Lengthening with external fixator; Epiphysiodesis
10	10	F	Achondroplasia	Lengthening with external fixator	None
11	8	F	Achondroplasia	None	None

**Table 2:** Key points raised during CDIs. * These participants needed two sessions to complete the CDIs. ** Due to children’s remarks 3 instructions and 11 items were changed.

Child	Key Points Raised During Cognitive Debriefing Interviews						
	I. Text Too Long or Lacks Clarity	II. Level of Language Too High	III. Lack of Cultural Familiarity	IV. Duplication of Descriptors	V. Use of Male/Female Endings	VI. Use of Vowel Markings	Too Many Items
1	✓	✓	✓			✓	
2	✓	✓					
3	✓	✓		✓			
4	✓	✓	✓				
5	✓	✓		✓	✓		
6	✓	✓	✓	✓			
7	✓	✓	✓				
8		✓	✓				
9	✓	✓	✓		✓	✓	✓ *
10	✓	✓	✓			✓	
11	✓	✓			✓	✓	✓ *
# text items changed **	3	5	4	2	Applies to all text		

## Data Availability

The original contributions presented in the study are included in the article/Supplementary Materials; further inquiries can be directed to the corresponding author.

## Supplementary Materials

The following supporting information can be downloaded at https://www.mdpi.com/article/10.3390/children12101318/s1; Figure S1: Lower limbs with labeled joints and segments. Document S2: Hebrew LIMB-Q KIDS Questionnaire.
