# Supplementary material for: Unique Challenges of Hebrew Translation and Cross-Cultural Adaptation of LIMB-Q Kids for Children with Lower Limb Differences
Source: Children (Basel). 2025 Oct 1;12(10):1318. doi: 10.3390/children12101318 (PMC12563977; doi:10.3390/children12101318)
Supplement: Supplementary file 1 [file children-12-01318-s001.zip › Document S2 Hebrew LIMB-Q KIDS Questionnaire .pdf]

LIMB-Q KIDS™ - נִרְאוּת (מַרְאֶה הָרַגְלַיִם)

אֵיךְ נִרְאֵית הָרֶגֶל שֶׁלָּךְ? **אָנָּא הָשִׁיבִי בְּהִתְיַיחֵס לְאֵיךְ שֶׁהָרֶגֶל שֶׁלָּךְ נִרְאֵית כָּרֶגַע.**

שְׁאֵלוֹת אֵלּוּ מִתְיַיחֲסוֹת לָרֶגֶל שֶׁבִּגְלָלָהּ אַתְּ הוֹלֶכֶת לָרוֹפֵא.

אִם אַתְּ מִשְׁתַּמֶּשֶׁת בְּאֲבִיזָר כְּמוֹ נַעַל עִם הַגְבָּהָה, מִדְרָס אוֹ סַד אוֹ רֶגֶל תּוֹתֶבֶת, תַּעֲנִי עַל הַשְּׁאֵלוֹת תּוֹךְ מַחֲשָׁבָה עַל הַזְּמַן בּוֹ אַתְּ מִשְׁתַּמֶּשֶׁת בְּאֲבִיזָר זֶה.

כַּמָּה אַתְּ אוֹהֶבֶת...	**בִּכְלָל לֹא**	**קְצָת**	**דֵּי הַרְבֵּה**	**מְאוֹד**
1. ... אֵיךְ שֶׁהָרֶגֶל שֶׁלָּךְ נִרְאֵית כְּשֶׁאַתְּ לוֹבֶשֶׁת מִכְנָסַיִם אֲרוּכִּים אוֹ ג'ִינְס?	1	2	3	4
2. ... אֵיךְ שֶׁהָרֶגֶל שֶׁלָּךְ נִרְאֵית כַּאֲשֶׁר אַתְּ יוֹשֶׁבֶת עַל כִּסֵּא?	1	2	3	4
3. ... אֵיךְ שֶׁהָרֶגֶל שֶׁלָּךְ נִרְאֵית?	1	2	3	4
4. ... אֵיךְ שֶׁהַבֶּרֶךְ שֶׁלָּךְ נִרְאֵית?	1	2	3	4
5. ... אֹרֶךְ הָרֶגֶל שֶׁלָּךְ?	1	2	3	4
6. ... גֹּדֶל הָרֶגֶל שֶׁלָּךְ?	1	2	3	4
7. ... אֵיךְ שֶׁהָרֶגֶל שֶׁלָּךְ נִרְאֵית כְּשֶׁאַתְּ לוֹבֶשֶׁת מִכְנָסַיִם קְצָרִים אוֹ חֲצָאִית?	1	2	3	4
8. ... כַּמָּה שֶׁהָרֶגֶל שֶׁלָּךְ נִרְאֵית יְשָׁרָה?	1	2	3	4
9. ... שֶׁהָרַגְלַיִם שֶׁלָּךְ דּוֹמוֹת אַחַת לִשְׁנִיָּה (נִרְאוֹת אוֹתוֹ הַדָּבָר)?	1	2	3	4
10. ...אֵיךְ שֶׁהָרֶגֶל שֶׁלָּךְ נִרְאֵית בְּהַשְׁוָאָה לְרַגְלַיִם שֶׁל אֲנָשִׁים אֲחֵרִים?	1	2	3	4

LIMB-Q KIDS™- צַלָּקוֹת

הַאִם יֵשׁ לָךְ צַלָּקוֹת מִנִּתּוּחַ עַל הָרֶגֶל שֶׁלָּךְ? אָנָּא הָשִׁיבִי בְּהִתְיַיחֵס לְאֵיךְ הַצַּלָּקוֹת שֶׁלָּךְ **<u>נִרְאוֹת כָּעֵת</u>.**

שְׁאֵלָה זוֹ מִתְיַיחֶסֶת לָרֶגֶל שֶׁבִּגְלָלָהּ אַתְּ הוֹלֶכֶת לָרוֹפֵא.

כַּמָּה אַתְּ אוֹהֶבֶת...	**בִּכְלָל לֹא**	**קְצָת**	**לְמַדַּי**	**מְאוֹד**
אֵיךְ שֶׁנִּרְאוֹת הַצַּלָּקוֹת עַל רַגְלֵךְ?	1	2	3	4

LIMB-Q KIDS™- תִּפְקוּד גּוּפָנִי

עַד כַּמָּה קַל לָךְ לְהִשְׁתַּמֵּשׁ בָּרֶגֶל שֶׁלָּךְ? תַּעֲנִי בַּמַּחְשָׁבָה עַל הַשָּׁבוּעַ הָאַחֲרוֹן.

בְּבַקָּשָׁה תַּעֲנִי לְגַבֵּי הָרֶגֶל שֶׁבִּגְלָלָהּ אַתְּ הוֹלֶכֶת לָרוֹפֵא.

אִם אַתְּ מִשְׁתַּמֶּשֶׁת בְּאֲבִיזָר כְּמוֹ נַעַל עִם הַגְבָּהָה, מִדְרָס אוֹ סַד אוֹ רֶגֶל תּוֹתֶבֶת, תַּעֲנִי עַל הַשְּׁאֵלוֹת תּוֹךְ מַחֲשָׁבָה עַל הַזְּמַן בּוֹ אַתְּ מִשְׁתַּמֶּשֶׁת בְּאֲבִיזָר זֶה.

הַאִם אַתְּ יְכוֹלָה לַעֲשׂוֹת אֶת הַדְּבָרִים הַבָּאִים:	**אֲנִי לֹא יְכוֹלָה לַעֲשׂוֹת אֶת זֶה**	**קָשֶׁה לִי לַעֲשׂוֹת אֶת זֶה**	**אֲנִי יְכוֹלָה לַעֲשׂוֹת אֶת זֶה**
1. ...לְהָרִים סֵפֶר מֵהָרִצְפָּה?	1	2	3
2. ...לָלֶכֶת תּוֹךְ הַחְזָקַת סֵפֶר בַּיָּדַיִים?	1	2	3
3. ...לָקוּם מֵרִצְפָּה	1	2	3
4. ... לָרֶדֶת בַּמַּדְרֵגוֹת?	1	2	3
5. ... לַעֲלוֹת בַּמַּדְרֵגוֹת?	1	2	3
6. ... לִקְפֹּץ?	1	2	3
7. ... לָלֶכֶת מַהֵר עַד כַּמָּה שֶׁאַתְּ רוֹצָה?	1	2	3
8. ...... לְטַפֵּס (לְמָשָׁל עַל סֻלָּם)?	1	2	3
9. ... לַעֲסֹק בִּסְפּוֹרְט שֶׁאַתְּ אוֹהֶבֶת?	1	2	3
10. ... לָלֶכֶת רָחוֹק עַד כַּמָּה שֶׁאַתְּ רוֹצָה?	1	2	3
11. ... לָרוּץ הֲכִי מַהֵר שֶׁאַתְּ רוֹצָה?	1	2	3

LIMB-Q-KIDS™- תַּסְמִינִים: רֶגֶל

אֵיךְ הָרֶגֶל שֶׁלָּךְ מַרְגִּישָׁה? אָנָּא הָשִׁיבִי בְּהִתְיַיחֵס לַשָּׁבוּעַ הָאַחֲרוֹן.

שְׁאֵלוֹת אֵלּוּ מִתְיַיחֲסוֹת לָרֶגֶל שֶׁבִּגְלָלָהּ אַתְּ הוֹלֶכֶת לָרוֹפֵא.

	תָּמִיד	לִפְעָמִים	אַף פַּעַם
1. כּוֹאֶבֶת לִי הָרֶגֶל כְּשֶׁאֲנִי יְשֵׁנָה.	1	2	3
2. כּוֹאֶבֶת לִי הָרֶגֶל כְּשֶׁאֲנִי נוֹגַעַת בָּהּ.	1	2	3
3. הָרֶגֶל שֶׁלִּי כּוֹאֶבֶת בִּזְמַן מְנוּחָה.	1	2	3
4. הָרֶגֶל שֶׁלִּי מַרְגִּישָׁה חַלָּשָׁה (רוֹעֶדֶת).	1	2	3
5. כּוֹאֶבֶת לִי הָרֶגֶל כְּשֶׁאֲנִי רָצָה.	1	2	3
6. כּוֹאֶבֶת לִי הָרֶגֶל כְּשֶׁאֲנִי הוֹלֶכֶת.	1	2	3
7. כּוֹאֶבֶת לִי הָרֶגֶל כְּשֶׁאֲנִי עוֹמֶדֶת זְמַן רַב.	1	2	3
8. הָרֶגֶל שֶׁלִּי מִתְעַיֶּיפֶת כַּאֲשֶׁר אֲנִי רָצָה.	1	2	3

LIMB-Q-KIDS™- תַּסְמִינִים: בֶּרֶךְ

אֵיךְ הַבֶּרֶךְ שֶׁלָּךְ מַרְגִּישָׁה? אָנָּא הָשִׁיבִי בְּהִתְיַיחֵס לַשָּׁבוּעַ הָאַחֲרוֹן.

שְׁאֵלוֹת אֵלּוּ מִתְיַיחֲסוֹת לְבֶּרֶךְ שֶׁבִּגְלָלָהּ אַתְּ הוֹלֶכֶת לָרוֹפֵא.

	תָּמִיד	לִפְעָמִים	אַף פַּעַם
1. הַבֶּרֶךְ שֶׁלִּי נִתְקַעַת (אֲנִי לֹא מְסֻגֶּלֶת לְהָזִיז אוֹתָהּ).	1	2	3
2. הַבֶּרֶךְ שֶׁלִּי כּוֹאֶבֶת בִּזְמַן מְנוּחָה.	1	2	3
3. הַבֶּרֶךְ שֶׁלִּי נְפוּחָה.	1	2	3
4. הַבֶּרֶךְ שֶׁלִּי מַרְגִּישָׁה חַלָּשָׁה (רוֹעֶדֶת).	1	2	3
5. הַבֶּרֶךְ שֶׁלִּי מַרְגִּישָׁה לֹא יַצִּיבָה (כְּאִלּוּ הִיא הוֹלֶכֶת לִקְרֹס).	1	2	3
6. הַבֶּרֶךְ שֶׁלִּי כּוֹאֶבֶת כַּאֲשֶׁר אֲנִי מְיַשֶּׁרֶת אוֹתָהּ.	1	2	3
7. הַבֶּרֶךְ שֶׁלִּי כּוֹאֶבֶת כַּאֲשֶׁר אֲנִי עוֹמֶדֶת הַרְבֵּה זְמַן.	1	2	3
8. הַבֶּרֶךְ שֶׁלִּי כּוֹאֶבֶת בִּזְמַן רִיצָה	1	2	3

תַּסְמִינִים: קַרְסוֹל וְכַף רֶגֶל -LIMB-Q-KIDS™

<u>אֵיךְ מַרְגִּישִׁים הַקַּרְסוֹל וְכַף הָרֶגֶל שֶׁלָּךְ?</u> אָנָּא הָשִׁיבִי בְּהִתְיַיחֵס <u>לַשָּׁבוּעַ הָאַחֲרוֹן.</u>

שְׁאֵלוֹת אֵלּוּ מִתְיַיחֲסוֹת לָרֶגֶל שֶׁבִּגְלָלָהּ אַתְּ הוֹלֶכֶת לָרוֹפֵא.

	תָּמִיד	לִפְעָמִים	אַף פַּעַם
1. הַקַּרְסוֹל שֶׁלִּי כּוֹאֵב בִּזְמַן מְנוּחָה.	1	2	3
2. כַּף הָרֶגֶל שֶׁלִּי כּוֹאֶבֶת בִּזְמַן מְנוּחָה.	1	2	3
3. הַקַּרְסוֹל שֶׁלִּי מַרְגִּישׁ לֹא יַצִּיב (כְּאִלּוּ הוּא הוֹלֵךְ לִקְרֹס).	1	2	3
4. הַקַּרְסוֹל שֶׁלִּי נִתְקַע (אֲנִי לֹא מְסֻגֶּלֶת לְהָזִיז אוֹתוֹ).	1	2	3
5. כַּף הָרֶגֶל שֶׁלִּי כּוֹאֶבֶת בִּזְמַן הֲלִיכָה עִם נַעֲלַיִם.	1	2	3
6. כַּף הָרֶגֶל שֶׁלִּי כּוֹאֶבֶת בִּזְמַן הֲלִיכָה בְּרַגְלַיִם יְחֵפוֹת (לְלֹא נַעֲלַיִם עַל הָרַגְלַיִם).	1	2	3
7. הַקַּרְסוֹל שֶׁלִּי כּוֹאֵב כְּשֶׁאֲנִי עוֹמֶדֶת הַרְבֵּה זְמַן.	1	2	3
8. הַקַּרְסוֹל שֶׁלִּי כּוֹאֵב בִּזְמַן רִיצָה.	1	2	3
9. כַּף הָרֶגֶל שֶׁלִּי כּוֹאֶבֶת בִּזְמַן רִיצָה.	1	2	3
10. כַּף הָרֶגֶל שֶׁלִּי כּוֹאֶבֶת כְּשֶׁאֲנִי עוֹמֶדֶת זְמַן רַב.	1	2	3

תְּחוּשַׁת מְצוּקָה הַקְּשׁוּרָה לָרֶגֶל -LIMB-Q-KIDS™

<u>אֵיךְ אַתְּ מַרְגִּישָׁה לְגַבֵּי הָרֶגֶל שֶׁלָּךְ? אָנָּא הָשִׁיבִי בְּהִתְיַיחֵס לַשָּׁבוּעַ הָאַחֲרוֹן.</u>

שְׁאֵלוֹת אֵלּוּ מִתְיַיחֲסוֹת לָרֶגֶל שֶׁבִּגְלָלָהּ אַתְּ הוֹלֶכֶת לָרוֹפֵא.

אִם אַתְּ מִשְׁתַּמֶּשֶׁת בְּאֲבִיזָר כְּמוֹ נַעַל עִם הַגְבָּהָה, מִדְרָס אוֹ סַד אוֹ רֶגֶל תּוֹתֶבֶת, תַּעֲנִי עַל הַשְּׁאֵלוֹת תּוֹךְ מַחֲשָׁבָה עַל הַזְּמַן בּוֹ אַתְּ מִשְׁתַּמֶּשֶׁת בְּאֲבִיזָר זֶה.

	תָּמִיד	לִפְעָמִים	אַף פַּעַם
1. אֲנִי נִמְנַעַת מִלָּצֵאת בִּגְלַל הָרֶגֶל שֶׁלִּי (לְמָשָׁל לִמְסִיבָּה).	1	2	3
2. אֲנִי מַסְתִּירָה אֶת הָרֶגֶל שֶׁלִּי כְּשֶׁאֲנִי יוֹצֵאת.	1	2	3
3. אֲנִי מִתְעַצְבֶּנֶת כַּאֲשֶׁר אֲנָשִׁים מִסְתַּכְּלִים עַל הָרֶגֶל שֶׁלִּי.	1	2	3
4. אֲנִי מִתְעַצְבֶּנֶת כַּאֲשֶׁר אֲנָשִׁים שׁוֹאֲלִים עַל הָרֶגֶל שֶׁלִּי.	1	2	3
5. אֲנִי נִמְנַעַת מִלִּלְבֹּשׁ מִכְנָסַיִם קְצָרִים אוֹ חֲצָאִיּוֹת שֶׁמַּרְאוֹת אֶת הָרֶגֶל שֶׁלִּי.	1	2	3
6. אֲנִי מַרְגִּישָׁה לֹא מְרֻצָּה לְגַבֵּי הָרֶגֶל שֶׁלִּי.	1	2	3
7. אֲנִי לֹא אוֹהֶבֶת אֵיךְ שֶׁאֲנִי נִרְאֵית כְּשֶׁאֲנִי הוֹלֶכֶת.	1	2	3
8. אֲנִי לֹא אוֹהֶבֶת אֵיךְ שֶׁהָרֶגֶל שֶׁלִּי נִרְאֵית.	1	2	3
9. הָרֶגֶל שֶׁלִּי גּוֹרֶמֶת לִי לְהַרְגִּישׁ שׁוֹנָה מֵאֲנָשִׁים אֲחֵרִים.	1	2	3
10. אֲנִי מֻדְאֶגֶת לְגַבֵּי הָרֶגֶל שֶׁלִּי.	1	2	3
11. אֲנִי מִתְעַצְבֶּנֶת כַּאֲשֶׁר הָרֶגֶל שֶׁלִּי מוֹנַעַת מִמֶּנִּי לֵהָנוֹת.	1	2	3

LIMB-Q-KIDS™ - תִּפְקוּד רִגְשִׁי

<u>אֵיךְ אַתְּ מַרְגִּישָׁה?</u> אָנָּא הָשִׁיבִי בְּהִתְיַיחֵס <u>לַשָּׁבוּעַ הָאַחֲרוֹן.</u>

	בִּכְלָל לֹא	לִפְעָמִים	לְעִתִּים קְרוֹבוֹת	תָּמִיד
1. אֲנִי נֶהֱנֵית מֵהַחַיִּים.	1	2	3	4
2. אֲנִי מַרְגִּישָׁה מְאֻשֶּׁרֶת.	1	2	3	4
3. אֲנִי מְרוּצָּה מֵהַחַיִּים שֶׁלִּי.	1	2	3	4
4. אֲנִי אוֹהֶבֶת אֶת עַצְמִי.	1	2	3	4
5. אֲנִי מַרְגִּישָׁה בְּסֵדֶר עִם עַצְמִי.	1	2	3	4
6. אֲנִי מַאֲמִינָה בְּעַצְמִי.	1	2	3	4
7. אֲנִי גֵּאָה בְּעַצְמִי.	1	2	3	4
8. אֲנִי מַרְגִּישָׁה בְּטוּחָה בְּעַצְמִי.	1	2	3	4
9. אֲנִי מַרְגִּישָׁה טוֹב עִם אֵיךְ שֶׁאֲנִי נִרְאֵית.	1	2	3	4

תִּפְקוּד חֶבְרָתִי – LIMB-Q-KIDS™

<u>אֵיךְ הַחַיִּים הַחֶבְרָתִיִּים שֶׁלָּךְ?</u> אָנָּא הָשִׁיבִי בְּהִתְיַיחֵס <u>לַשָּׁבוּעַ הָאַחֲרוֹן.</u>

	**בִּכְלָל לֹא**	**לִפְעָמִים**	**לְעִתִּים קְרוֹבוֹת**	**תָּמִיד**
1. כֵּיף לִי עִם חֲבֵרַי.	1	2	3	4
2. אֲנִי מְקֻבֶּלֶת עַל חֲבֵרַי.	1	2	3	4
3. אֲנָשִׁים מַקְשִׁיבִים לְמָה שֶׁיֵּשׁ לִי לְהַגִּיד.	1	2	3	4
4. אֲנָשִׁים מִתְיַיחֲסִים אֵלַי כְּמוֹ לְכוּלָּם.	1	2	3	4
5. אֲנִי אוֹהֶבֶת לִהְיוֹת עִם אֲנָשִׁים אֲחֵרִים.	1	2	3	4
6. אֲנִי מַרְגִּישָׁה שֶׁאֲנִי מִשְׁתַּלֶּבֶת.	1	2	3	4
7. קַל לִי לְהַכִּיר חֲבֵרִים.	1	2	3	4
8. אֲנִי מַרְגִּישָׁה בְּטוּחָה בְּעַצְמִי כְּשֶׁאֲנִי יוֹצֵאת (לְמָשָׁל לִמְסִבָּה).	1	2	3	4
9. חֲבֵרִים מְבַקְשִׁים מִמֶּנִּי לָצֵאת אִתָּם.	1	2	3	4
10. אֲנִי מַרְגִּישָׁה כְּמוֹ אֲנָשִׁים אֲחֵרִים בְּגִילִי.	1	2	3	4

LIMB-Q KIDS™ - נראות (מראה הרגליים)

איך נראית הרגל שלך? אנא השיבי בהתייחס לאיך שהרגל שלך נראית כרגע.

שאלות אלו מתייחסות לרגל שבגללה את הולכת לרופא.

אם את משתמשת באביזר כמו נעל עם הַגְבָּהָה, מִדְרָס או סד או רגל תותבת, תעני על השאלות תוך מחשבה על הזמן בו את משתמשת באביזר זה.

כמה את אוהבת...	**בכלל לא**	**קצת**	**די הרבה**	**מאוד**
1. ... איך שהרגל שלך נראית כשאת לובשת מכנסיים ארוכים או ג'ינס?	1	2	3	4
2. ... איך שהרגל שלך נראית כאשר את יושבת על כיסא?	1	2	3	4
3. ... איך שהרגל שלך נראית?	1	2	3	4
4. ... איך שהברך שלך נראית?	1	2	3	4
5. ... אורך הרגל שלך?	1	2	3	4
6. ... גודל הרגל שלך?	1	2	3	4
7. ...איך שהרגל שלך נראית כשאת לובשת מכנסיים קצרים או חצאית?	1	2	3	4
8. ...כמה שהרגל שלך נראית ישרה?	1	2	3	4
9. ... את כמה שהרגליים שלך דומות אחת לשנייה (נראות אותו הדבר)?	1	2	3	4
10. ...איך שהרגל שלך נראית בהשוואה לרגליים של אנשים אחרים?	1	2	3	4

LIMB-Q KIDS™- צלקות

האם יש לך צלקות מניתוח על הרגל שלך? **אנא השיבי בהתייחס לאיך הצלקות שלך <u>נראות כעת</u>.**

שאלה זו מתייחסת לרגל שבגללה את הולכת לרופא.

כמה את אוהבת...	**בכלל לא**	**קצת**	**למדיי**	**מאוד**
איך שנראות הצלקות על רגלך?	1	2	3	4

תפקוד גופני -LIMB-Q KIDS™

עד כמה קל לך להשתמש ברגל שלך? תעני במחשבה על השבוע האחרון.

בבקשה תעני לגבי הרגל שבגללה את הולכת לרופא.

אם את משתמשת באביזר כמו נעל עם הַגְבָּהָה, מִדְרָס או סד או רגל תותבת, תעני על השאלות תוך מחשבה על הזמן בו את משתמשת באביזר זה.

האם את יכולה לעשות את הדברים הבאים:	**אני לא יכולה לעשות את זה**	**קשה לי לעשות את זה**	**אני יכולה לעשות את זה**
1. ...להרים ספר מהרצפה?	1	2	3
2. ...ללכת תוך החזקת ספר בידיים?	1	2	3
3. ...לקום מרצפה	1	2	3
4. ... לרדת במדרגות?	1	2	3
5. ... לעלות במדרגות?	1	2	3
6. ... לקפוץ?	1	2	3
7. ... ללכת מהר עד כמה שאת רוצה?	1	2	3
8...... לטפס (למשל על סולם)?	1	2	3
9. ... לעסוק בספורט שאני אוהבת?	1	2	3
10. ... ללכת רחוק עד כמה שאתה רוצה?	1	2	3
11. ... לרוץ הכי מהר שאת רוצה?	1	2	3

LIMB-Q-KIDS™- תסמינים: רגל

איך הרגל שלך מרגישה? אנא השיבי בהתייחס <u>לשבוע האחרון</u>.

שאלות אלו מתייחסות לרגל שבגללה את הולכת לרופא.

	תמיד	לפעמים	אף פעם
1. כואבת לי הרגל כשאני ישנה.	1	2	3
2. כואבת לי הרגל כשאני נוגעת בה.	1	2	3
3. הרגל שלי כואבת בזמן מנוחה.	1	2	3
4. הרגל שלי מרגישה חלשה (רועדת).	1	2	3
5. כואבת לי הרגל כשאני רצה.	1	2	3
6. כואבת לי הרגל כשאני הולכת.	1	2	3
7. כואבת לי הרגל כשאני עומדת זמן רב.	1	2	3
8. הרגל שלי מתעייפת כאשר אני רצה.	1	2	3

LIMB-Q-KIDS™- תסמינים: ברך

<u>איך הברך שלך מרגישה?</u> אנא השיבי בהתייחס <u>לשבוע האחרון</u>.

שאלות אלו מתייחסות לברך שבגללה את הולכת לרופא.

	תמיד	לפעמים	אף פעם
1. הברך שלי נתקעת (אני לא מסוגלת להזיז אותה).	1	2	3
2. הברך שלי כואבת בזמן מנוחה.	1	2	3
3. הברך שלי נפוחה.	1	2	3
4. הברך שלי מרגישה חלשה (רועדת).	1	2	3
5. הברך שלי מרגישה לא יציבה (כאילו היא הולכת לקרוס).	1	2	3
6. הברך שלי כואבת כאשר אני מיישרת אותה.	1	2	3
7. הברך שלי כואבת כאשר אני עומדת הרבה זמן.	1	2	3
8. הברך שלי כואבת בזמן ריצה	1	2	3

LIMB-Q-KIDS™- תסמינים: קרסול וכף רגל

<u>איך מרגישים הקרסול וכף הרגל שלך?</u> אנא השיבי בהתייחס <u>לשבוע האחרון.</u>

שאלות אלו מתייחסות לרגל שבגללה את הולכת לרופא.

	תמיד	לפעמים	אף פעם
1. הקרסול שלי כואב בזמן מנוחה.	1	2	3
2. כף הרגל שלי כואבת בזמן מנוחה.	1	2	3
3. הקרסול שלי מרגיש לא יציב (כאילו הוא הולך לְקְרֹס).	1	2	3
4. הקרסול שלי נתקע (אני לא מסוגלת להזיז אותו).	1	2	3
5. כף הרגל שלי כואבת בעת הליכה עם נעליים.	1	2	3
6. כף הרגל שלי כואבת בעת הליכה ברגליים יחפות (ללא נעליים על הרגליים).	1	2	3
7. הקרסול שלי כואב כשאני עומדת הרבה זמן.	1	2	3
8. הקרסול שלי כואב בעת ריצה.	1	2	3
9. כף הרגל שלי כואבת בעת ריצה.	1	2	3
10. כף הרגל שלי כואבת כשאני עומדת זמן רב.	1	2	3

LIMB-Q-KIDS™- תחושת מצוקה הקשורה לרגל

איך את מרגישה לגבי הרגל שלך? אנא השיבי בהתייחס לשבוע האחרון.

שאלות אלו מתייחסות לרגל שבגללה את הולכת לרופא.

אם את משתמשת באביזר כמו נעל עם הַגְבָּהָה, מִדְרָס או סד או רגל תותבת, תעני על השאלות תוך מחשבה על הזמן בו את משתמשת באביזר זה.

	תמיד	לפעמים	אף פעם
1. אני נמנעת מלצאת בגלל הרגל שלי (למשל למסיבה).	1	2	3
2. אני מסתירה את הרגל שלי כשאני יוצאת.	1	2	3
3. אני מתעצבנת כאשר אנשים מסתכלים על הרגל שלי.	1	2	3
4. אני מתעצבנת כאשר אנשים שואלים על הרגל שלי.	1	2	3
5. אני נמנעת מללבוש מכנסיים קצרים או חצאיות שמראות את הרגל שלי.	1	2	3
6. אני מרגישה לא מרוצה לגבי הרגל שלי.	1	2	3
7. אני לא אוהבת איך שאני נראית כשאני הולכת.	1	2	3
8. אני לא אוהבת איך שהרגל שלי נראית.	1	2	3
9. הרגל שלי גורמת לי להרגיש שונה מאנשים אחרים.	1	2	3
10. אני מודאגת לגבי הרגל שלי.	1	2	3
11. אני מתעצבנת כאשר הרגל שלי מונעת ממני ליהנות.	1	2	3

תפקוד רגשי - LIMB-Q-KIDS™

איך את מרגישה? אנא השיבי בהתייחס לשבוע האחרון.

	בכלל לא	לפעמים	לעתים קרובות	תמיד
1. אני נהנית מהחיים.	1	2	3	4
2. אני מרגישה מאושרת.	1	2	3	4
3. אני מרוצה מהחיים שלי.	1	2	3	4
4. אני אוהבת את עצמי.	1	2	3	4
5. אני מרגישה בסדר עם עצמי.	1	2	3	4
6. אני מאמינה בעצמי.	1	2	3	4
7. אני גאה בעצמי.	1	2	3	4
8. אני מרגישה בטוחה בעצמי.	1	2	3	4
9. אני מרגישה טוב עם איך שאני נראית.	1	2	3	4

תפקוד חברתי – LIMB-Q-KIDS™

איך החיים החברתיים שלך? אנא השיבי בהתייחס לשבוע האחרון.

	בכלל לא	לפעמים	לעתים קרובות	תמיד
1. כיף לי עם חבריי.	1	2	3	4
2. אני מקובלת על חבריי.	1	2	3	4
3. אנשים מקשיבים למה שיש לי להגיד.	1	2	3	4
4. אנשים מתייחסים אלי כמו לכולם.	1	2	3	4
5. אני אוהבת להיות עם אנשים אחרים.	1	2	3	4
6. אני מרגישה שאני משתלבת.	1	2	3	4
7. קל לי להכיר חברים.	1	2	3	4
8. אני מרגישה בטוחה בעצמי כשאני יוצאת (למשל למסיבה).	1	2	3	4
9. חברים מבקשים ממני לצאת איתם.	1	2	3	4
10. אני מרגישה כמו אנשים אחרים בגילי.	1	2	3	4

נִרְאוּת (מַרְאֶה הָרַגְלַיִם)- LIMB-Q KIDS™

אֵיךְ נִרְאֵית הָרֶגֶל שֶׁלְּךָ? אָנָּא הָשֵׁב בְּהִתְיַיחֵס לְאֵיךְ שֶׁהָרֶגֶל שֶׁלְּךָ **נִרְאֵית כָּרֶגַע.**

שְׁאֵלוֹת אֵלּוּ מִתְיַיחֲסוֹת לָרֶגֶל שֶׁבִּגְלָלָהּ אַתָּה הוֹלֵךְ לְרוֹפֵא.

אִם אַתָּה מִשְׁתַּמֵּשׁ בְּאֲבִיזָר כְּמוֹ נַעַל עִם הַגְבָּהָה, מִדְרָס אוֹ סַד אוֹ רֶגֶל תּוֹתֶבֶת, תַּעֲנֶה עַל הַשְּׁאֵלוֹת תּוֹךְ מַחְשָׁבָה עַל הַזְּמַן בּוֹ אַתָּה מִשְׁתַּמֵּשׁ בְּאֲבִיזָר זֶה.

כַּמָּה אַתָּה אוֹהֵב...	בִּכְלָל לֹא	קְצָת	דֵּי הַרְבֵּה	מְאוֹד
1. ... אֵיךְ שֶׁהָרֶגֶל שֶׁלְּךָ נִרְאֵית כְּשֶׁאַתָּה לוֹבֵשׁ מִכְנָסַיִם אֲרֻכִּים אוֹ גִ'ינְס?	1	2	3	4
2. ... אֵיךְ שֶׁהָרֶגֶל שֶׁלְּךָ נִרְאֵית כַּאֲשֶׁר אַתָּה יוֹשֵׁב עַל כִּסֵּא?	1	2	3	4
3. ... אֵיךְ שֶׁהָרֶגֶל שֶׁלְּךָ נִרְאֵית?	1	2	3	4
4. ... אֵיךְ שֶׁהַבֶּרֶךְ שֶׁלְּךָ נִרְאֵית?	1	2	3	4
5. ... אֹרֶךְ הָרֶגֶל שֶׁלְּךָ?	1	2	3	4
6. ... גֹּדֶל הָרֶגֶל שֶׁלְּךָ?	1	2	3	4
7. ...אֵיךְ שֶׁהָרֶגֶל שֶׁלְּךָ נִרְאֵית כְּשֶׁאַתָּה לוֹבֵשׁ מִכְנָסַיִם קְצָרִים?	1	2	3	4
8. ...כַּמָּה שֶׁהָרֶגֶל שֶׁלְּךָ נִרְאֵית יְשָׁרָה?	1	2	3	4
9. ... שֶׁהָרַגְלַיִם שֶׁלְּךָ דּוֹמוֹת אַחַת לִשְׁנִיָּה (נִרְאוֹת אוֹתוֹ הַדָּבָר)?	1	2	3	4
10. ...אֵיךְ שֶׁהָרֶגֶל שֶׁלְּךָ נִרְאֵית בְּהַשְׁוָאָה לְרַגְלַיִם שֶׁל אֲנָשִׁים אֲחֵרִים?	1	2	3	4

צַלָּקוֹת- LIMB-Q KIDS™

הַאִם יֵשׁ לְךָ צַלָּקוֹת מִנִּתּוּחַ עַל הָרֶגֶל שֶׁלְּךָ? אָנָּא הָשֵׁב בְּהִתְיַיחֵס לְאֵיךְ הַצַּלָּקוֹת שֶׁלְּךָ **נִרְאוֹת כָּעֵת.**

שְׁאֵלָה זוֹ מִתְיַיחֶסֶת לָרֶגֶל שֶׁבִּגְלָלָהּ אַתָּה הוֹלֵךְ לְרוֹפֵא.

כַּמָּה אַתָּה אוֹהֵב...	**בִּכְלָל לֹא**	**קְצָת**	**לְמַדַּי**	**מְאוֹד**
אֵיךְ שֶׁנִּרְאוֹת הַצַּלָּקוֹת עַל רַגְלְךָ?	1	2	3	4

LIMB-Q KIDS™- תִּפְקוּד גּוּפָנִי

עַד כַּמָּה קַל לְךָ לְהִשְׁתַּמֵּשׁ בָּרֶגֶל שֶׁלְּךָ? תַּעֲנֶה בַּמַּחְשָׁבָה עַל הַשָּׁבוּעַ הָאַחֲרוֹן.

בְּבַקָּשָׁה תַּעֲנֶה לְגַבֵּי הָרֶגֶל שֶׁבִּגְלָלָהּ אַתָּה הוֹלֵךְ לָרוֹפֵא.

אִם אַתָּה מִשְׁתַּמֵּשׁ בְּאֲבִיזָר כְּמוֹ נַעַל עִם הַגְבָּהָה, מִדְרָס אוֹ סַד אוֹ רֶגֶל תּוֹתֶבֶת, תַּעֲנֶה עַל הַשְּׁאֵלוֹת תּוֹךְ מַחְשָׁבָה עַל הַזְּמַן בּוֹ אַתָּה מִשְׁתַּמֵּשׁ בְּאֲבִיזָר זֶה.

הַאִם אַתָּה יָכוֹל לַעֲשׂוֹת אֶת הַדְּבָרִים הַבָּאִים:	**אֲנִי לֹא יָכוֹל לַעֲשׂוֹת אֶת זֶה**	**קָשֶׁה לִי לַעֲשׂוֹת אֶת זֶה**	**אֲנִי יָכוֹל לַעֲשׂוֹת אֶת זֶה**
1. ...לְהָרִים סֵפֶר מֵהָרִצְפָּה?	1	2	3
2. ...לָלֶכֶת תּוֹךְ הַחְזָקַת סֵפֶר בַּיָּדַיִים?	1	2	3
3. ...לָקוּם מֵרִצְפָּה	1	2	3
4. ... לָרֶדֶת בַּמַּדְרֵגוֹת?	1	2	3
5. ... לַעֲלוֹת בַּמַּדְרֵגוֹת?	1	2	3
6. ... לִקְפֹּץ?	1	2	3
7. ... לָלֶכֶת מַהֵר עַד כַּמָּה שֶׁאַתָּה רוֹצֶה?	1	2	3
8. ......לְטַפֵּס (לְמָשָׁל עַל סֻלָּם)?	1	2	3
9. ... לַעֲסֹק בְּסְפּוֹרְט שֶׁאַתָּה אוֹהֵב?	1	2	3
10. ... לָלֶכֶת רָחוֹק עַד כַּמָּה שֶׁאַתָּה רוֹצֶה?	1	2	3
11. ... לָרוּץ הֲכִי מַהֵר שֶׁאַתָּה רוֹצֶה?	1	2	3

LIMB-Q-KIDS™- תַּסְמִינִים: רֶגֶל

אֵיךְ הָרֶגֶל שֶׁלְּךָ מַרְגִּישָׁה? אָנָּא הָשֵׁב בְּהִתְיַיחֵס לַשָּׁבוּעַ הָאַחֲרוֹן.

שְׁאֵלוֹת אֵלּוּ מִתְיַיחֲסוֹת לָרֶגֶל שֶׁבִּגְלָלָהּ אַתָּה הוֹלֵךְ לְרוֹפֵא.

	תָּמִיד	לִפְעָמִים	אַף פַּעַם
1. כּוֹאֶבֶת לִי הָרֶגֶל כְּשֶׁאֲנִי יָשֵׁן.	1	2	3
2. כּוֹאֶבֶת לִי הָרֶגֶל כְּשֶׁאֲנִי נוֹגֵעַ בָּהּ.	1	2	3
3. הָרֶגֶל שֶׁלִּי כּוֹאֶבֶת בִּזְמַן מְנוּחָה.	1	2	3
4. הָרֶגֶל שֶׁלִּי מַרְגִּישָׁה חַלָּשָׁה (רוֹעֶדֶת).	1	2	3
5. כּוֹאֶבֶת לִי הָרֶגֶל כְּשֶׁאֲנִי רָץ.	1	2	3
6. כּוֹאֶבֶת לִי הָרֶגֶל כְּשֶׁאֲנִי הוֹלֵךְ.	1	2	3
7. כּוֹאֶבֶת לִי הָרֶגֶל כְּשֶׁאֲנִי עוֹמֵד זְמַן רַב.	1	2	3
8. הָרֶגֶל שֶׁלִּי מִתְעַיֶּפֶת כַּאֲשֶׁר אֲנִי רָץ.	1	2	3

LIMB-Q-KIDS™- תַּסְמִינִים: בֶּרֶךְ

<u>אֵיךְ הַבֶּרֶךְ שֶׁלְּךָ מַרְגִּישָׁה?</u> אָנָּא הָשֵׁב בְּהִתְיַיחֵס <u>לַשָּׁבוּעַ הָאַחֲרוֹן.</u>

שְׁאֵלוֹת אֵלּוּ מִתְיַיחֲסוֹת לַבֶּרֶךְ שֶׁבִּגְלָלָהּ אַתָּה הוֹלֵךְ לָרוֹפֵא.

	תָּמִיד	לִפְעָמִים	אַף פַּעַם
1. הַבֶּרֶךְ שֶׁלִּי נִתְקַעַת (אֲנִי לֹא מְסֻגָּל לְהָזִיז אוֹתָהּ).	1	2	3
2. הַבֶּרֶךְ שֶׁלִּי כּוֹאֶבֶת בִּזְמַן מְנוּחָה.	1	2	3
3. הַבֶּרֶךְ שֶׁלִּי נְפוּחָה.	1	2	3
4. הַבֶּרֶךְ שֶׁלִּי מַרְגִּישָׁה חַלָּשָׁה (רוֹעֶדֶת).	1	2	3
5. הַבֶּרֶךְ שֶׁלִּי מַרְגִּישָׁה לֹא יַצִּיבָה (כְּאִלּוּ הִיא הוֹלֶכֶת לִקְרֹס).	1	2	3
6. הַבֶּרֶךְ שֶׁלִּי כּוֹאֶבֶת כַּאֲשֶׁר אֲנִי מְיַשֵּׁר אוֹתָהּ.	1	2	3
7. הַבֶּרֶךְ שֶׁלִּי כּוֹאֶבֶת כַּאֲשֶׁר אֲנִי עוֹמֵד הַרְבֵּה זְמַן.	1	2	3
8. הַבֶּרֶךְ שֶׁלִּי כּוֹאֶבֶת בִּזְמַן רִיצָה	1	2	3

LIMB-Q-KIDS™- תַּסְמִינִים: קַרְסוֹל וְכַף רֶגֶל

<u>אֵיךְ מַרְגִּישִׁים הַקַּרְסוֹל וְכַף הָרֶגֶל שֶׁלְּךָ?</u> אָנָּא הָשֵׁב בְּהִתְיַיחֵס <u>לַשָּׁבוּעַ הָאַחֲרוֹן.</u>

שְׁאֵלוֹת אֵלּוּ מִתְיַיחֲסוֹת לָרֶגֶל שֶׁבִּגְלָלָהּ אַתָּה הוֹלֵךְ לְרוֹפֵא.

	תָּמִיד	לִפְעָמִים	אַף פַּעַם
1. הַקַּרְסוֹל שֶׁלִּי כּוֹאֵב בִּזְמַן מְנוּחָה.	1	2	3
2. כַּף הָרֶגֶל שֶׁלִּי כּוֹאֶבֶת בִּזְמַן מְנוּחָה.	1	2	3
3. הַקַּרְסוֹל שֶׁלִּי מַרְגִּישׁ לֹא יַצִּיב (כְּאִלּוּ הוּא הוֹלֵךְ לְקְרֹס).	1	2	3
4. הַקַּרְסוֹל שֶׁלִּי נִתְקַע (אֲנִי לֹא מְסֻגָּל לְהָזִיז אוֹתוֹ).	1	2	3
5. כַּף הָרֶגֶל שֶׁלִּי כּוֹאֶבֶת בִּזְמַן הֲלִיכָה עִם נַעֲלַיִם.	1	2	3
6. כַּף הָרֶגֶל שֶׁלִּי כּוֹאֶבֶת בִּזְמַן הֲלִיכָה בְּרַגְלַיִם יְחֵפוֹת (לְלֹא נַעֲלַיִם עַל הָרַגְלַיִם).	1	2	3
7. הַקַּרְסוֹל שֶׁלִּי כּוֹאֵב כְּשֶׁאֲנִי עוֹמֵד הַרְבֵּה זְמַן.	1	2	3
8. הַקַּרְסוֹל שֶׁלִּי כּוֹאֵב בִּזְמַן רִיצָה.	1	2	3
9. כַּף הָרֶגֶל שֶׁלִּי כּוֹאֶבֶת בִּזְמַן רִיצָה.	1	2	3
10. כַּף הָרֶגֶל שֶׁלִּי כּוֹאֶבֶת כְּשֶׁאֲנִי עוֹמֵד זְמַן רַב.	1	2	3

תְּחוּשַׁת מְצוּקָה הַקְּשׁוּרָה לָרֶגֶל -LIMB-Q-KIDS™

<u>אֵיךְ אַתָּה מַרְגִּישׁ לְגַבֵּי הָרֶגֶל שֶׁלְּךָ?</u> אָנָּא הָשֵׁב <u>בְּהִתְיַיחֵס לַשָּׁבוּעַ הָאַחֲרוֹן.</u>

שְׁאֵלוֹת אֵלּוּ מִתְיַיחֲסוֹת לָרֶגֶל שֶׁבִּגְלָלָהּ אַתָּה הוֹלֵךְ לְרוֹפֵא.

אִם אַתָּה מִשְׁתַּמֵּשׁ בְּאֲבִיזָר כְּמוֹ נַעַל עִם הַגְבָּהָה, מִדְרָס אוֹ סַד אוֹ רֶגֶל תּוֹתֶבֶת, תַּעֲנֶה עַל הַשְּׁאֵלוֹת תּוֹךְ מַחְשָׁבָה עַל הַזְּמַן בּוֹ אַתָּה מִשְׁתַּמֵּשׁ בְּאֲבִיזָר זֶה.

	תָּמִיד	לִפְעָמִים	אַף פַּעַם
1. אֲנִי נִמְנָע מִלָּצֵאת בִּגְלַל הָרֶגֶל שֶׁלִּי (לְמָשָׁל לִמְסִבָּה).	1	2	3
2. אֲנִי מַסְתִּיר אֶת הָרֶגֶל שֶׁלִּי כְּשֶׁאֲנִי יוֹצֵא.	1	2	3
3. אֲנִי מִתְעַצְבֵּן כַּאֲשֶׁר אֲנָשִׁים מִסְתַּכְּלִים עַל הָרֶגֶל שֶׁלִּי.	1	2	3
4. אֲנִי מִתְעַצְבֵּן כַּאֲשֶׁר אֲנָשִׁים שׁוֹאֲלִים עַל הָרֶגֶל שֶׁלִּי.	1	2	3
5. אֲנִי נִמְנָע מִלִּלְבֹּשׁ מִכְנָסַיִם קְצָרִים שֶׁמַּרְאִים אֶת הָרֶגֶל שֶׁלִּי.	1	2	3
6. אֲנִי מַרְגִּישׁ לֹא מְרֻצֶּה לְגַבֵּי הָרֶגֶל שֶׁלִּי.	1	2	3
7. אֲנִי לֹא אוֹהֵב אֵיךְ שֶׁאֲנִי נִרְאֶה כְּשֶׁאֲנִי הוֹלֵךְ.	1	2	3
8. אֲנִי לֹא אוֹהֵב אֵיךְ שֶׁהָרֶגֶל שֶׁלִּי נִרְאֵית.	1	2	3
9. הָרֶגֶל שֶׁלִּי גּוֹרֶמֶת לִי לְהַרְגִּישׁ שׁוֹנֶה מֵאֲנָשִׁים אֲחֵרִים.	1	2	3
10. אֲנִי מֻדְאָג לְגַבֵּי הָרֶגֶל שֶׁלִּי.	1	2	3
11. אֲנִי מִתְעַצְבֵּן כַּאֲשֶׁר הָרֶגֶל שֶׁלִּי מוֹנַעַת מִמֶּנִּי לֵהָנוֹת.	1	2	3

LIMB-Q-KIDS™ - תִּפְקוּד רִגְשִׁי

איךְ אַתָּה מַרְגִּישׁ? אָנָּא הָשֵׁב בְּהִתְיַיחֵס לַשָּׁבוּעַ הָאַחֲרוֹן.

	בִּכְלָל לֹא	לִפְעָמִים	לְעִתִּים קְרוֹבוֹת	תָּמִיד
1. אֲנִי נֶהֱנֶה מֵהַחַיִּים.	1	2	3	4
2. אֲנִי מַרְגִּישׁ מְאֻשָּׁר.	1	2	3	4
3. אֲנִי מְרֻצֶּה מֵהַחַיִּים שֶׁלִּי.	1	2	3	4
4. אֲנִי אוֹהֵב אֶת עַצְמִי.	1	2	3	4
5. אֲנִי מַרְגִּישׁ בְּסֵדֶר עִם עַצְמִי.	1	2	3	4
6. אֲנִי מַאֲמִין בְּעַצְמִי.	1	2	3	4
7. אֲנִי גֵּאֶה בְּעַצְמִי.	1	2	3	4
8. אֲנִי מַרְגִּישׁ בָּטוּחַ בְּעַצְמִי.	1	2	3	4
9. אֲנִי מַרְגִּישׁ טוֹב עִם אֵיךְ שֶׁאֲנִי נִרְאֶה.	1	2	3	4

LIMB-Q-KIDS™ – תִּפְקוּד חֶבְרָתִי

איךְ הַחַיִּים הַחֶבְרָתִיִּים שֶׁלְּךָ? אָנָּא הָשֵׁב בְּהִתְיַיחֵס **לַשָּׁבוּעַ הָאַחֲרוֹן.**

	בִּכְלָל לֹא	לִפְעָמִים	לְעִתִּים קְרוֹבוֹת	תָּמִיד
1. כֵּיף לִי עִם חֲבֵרַי.	1	2	3	4
2. אֲנִי מְקֻבָּל עַל חֲבֵרַי.	1	2	3	4
3. אֲנָשִׁים מַקְשִׁיבִים לְמָה שֶׁיֵּשׁ לִי לְהַגִּיד.	1	2	3	4
4. אֲנָשִׁים מִתְיַיחֲסִים אֵלַי כְּמוֹ לְכֻלָּם.	1	2	3	4
5. אֲנִי אוֹהֵב לִהְיוֹת עִם אֲנָשִׁים אֲחֵרִים.	1	2	3	4
6. אֲנִי מַרְגִּישׁ שֶׁאֲנִי מִשְׁתַּלֵּב.	1	2	3	4
7. קַל לִי לְהַכִּיר חֲבֵרִים.	1	2	3	4
8. אֲנִי מַרְגִּישׁ בָּטוּחַ בְּעַצְמִי כְּשֶׁאֲנִי יוֹצֵא (לְמָשָׁל לִמְסִבָּה).	1	2	3	4
9. חֲבֵרִים מְבַקְּשִׁים מִמֶּנִּי לָצֵאת אִתָּם.	1	2	3	4
10. אֲנִי מַרְגִּישׁ כְּמוֹ אֲנָשִׁים אֲחֵרִים בְּגִילִי.	1	2	3	4

LIMB-Q KIDS™ - נראות (מראה הרגליים)

איך נראית הרגל שלך? **אנא השב בהתייחס לאיך שהרגל שלך נראית כרגע.**

שאלות אלו מתייחסות לרגל שבגללה אתה הולך לרופא.

אם אתה משתמש באביזר כמו נעל עם הַגְבָּהָה, מִדְרָס או סד או רגל תותבת, תענה על השאלות תוך מחשבה על הזמן בו אתה משתמש באביזר זה.

כמה אתה אוהב...	בכלל לא	קצת	די הרבה	מאוד
1. ... איך שהרגל שלך נראית כשאתה לובש מכנסיים ארוכים או ג'ינס?	1	2	3	4
2. ... איך שהרגל שלך נראית כאשר אתה יושב על כיסא?	1	2	3	4
3. ... איך שהרגל שלך נראית?	1	2	3	4
4. ... איך שהברך שלך נראית?	1	2	3	4
5. ... אורך הרגל שלך?	1	2	3	4
6. ... גודל הרגל שלך?	1	2	3	4
7. ...איך שהרגל שלך נראית כשאתה לובש מכנסיים קצרים או חצאית?	1	2	3	4
8. ...כמה שהרגל שלך נראית ישרה?	1	2	3	4
9. ... את כמה שהרגליים שלך דומות אחת לשנייה (נראות אותו הדבר)?	1	2	3	4
10. ...איך שהרגל שלך נראית בהשוואה לרגליים של אנשים אחרים?	1	2	3	4

צלקות -LIMB-Q KIDS™

האם יש לך צלקות מניתוח על הרגל שלך? אנא השב בהתייחס לאיך הצלקות שלך <u>נראות כעת</u>.

שאלה זו מתייחסת לרגל שבגללה אתה הולך לרופא.

כמה אתה אוהב...	**בכלל לא**	**קצת**	**למדיי**	**מאוד**
איך שנראות הצלקות על רגלך?	1	2	3	4

LIMB-Q KIDS™- תפקוד גופני

עד כמה קל לך להשתמש ברגל שלך? תענה במחשבה על השבוע האחרון.

בבקשה תענה לגבי הרגל שבגללה אתה הולך לרופא.

אם אתה משתמש באביזר כמו נעל עם הַגְבָּהָה, מִדְרָס או סד או רגל תותבת, תענה על השאלות תוך מחשבה על הזמן בו אתה משתמש באביזר זה.

האם אתה יכול לעשות את הדברים הבאים:	**אני לא יכול לעשות את זה**	**קשה לי לעשות את זה**	**אני יכול לעשות את זה**
1. ...להרים ספר מהרצפה?	1	2	3
2. ...ללכת תוך החזקת ספר בידיים?	1	2	3
3. ...לקום מרצפה	1	2	3
4. ... לרדת במדרגות?	1	2	3
5. ... לעלות במדרגות?	1	2	3
6. ... לקפוץ?	1	2	3
7. ... ללכת מהר עד כמה שאתה רוצה?	1	2	3
8...... לטפס (למשל על סולם)?	1	2	3
9. ... לעסוק בספורט שאתה אוהב?	1	2	3
10. ... ללכת רחוק עד כמה שאתה רוצה?	1	2	3
11. ... לרוץ הכי מהר שאתה רוצה?	1	2	3

LIMB-Q-KIDSTM- תסמינים: רגל

איך הרגל שלך מרגישה? אנא השב בהתייחס לשבוע האחרון.

שאלות אלו מתייחסות לרגל שבגללה אתה הולך לרופא.

	תמיד	לפעמים	אף פעם
1. כואבת לי הרגל כשאני ישן.	1	2	3
2. כואבת לי הרגל כשאני נוגע בה.	1	2	3
3. הרגל שלי כואבת בזמן מנוחה.	1	2	3
4. הרגל שלי מרגישה חלשה (רועדת).	1	2	3
5. כואבת לי הרגל כשאני רץ.	1	2	3
6. כואבת לי הרגל כשאני הולך.	1	2	3
7. כואבת לי הרגל כשאני עומד זמן רב.	1	2	3
8. הרגל שלי מתעייפת כאשר אני רץ.	1	2	3

LIMB-Q-KIDS™- תסמינים: ברך

<u>איך הברך שלך מרגישה?</u> אנא השב בהתייחס <u>לשבוע האחרון</u>.

שאלות אלו מתייחסות לברך שבגללה אתה הולך לרופא.

	תמיד	לפעמים	אף פעם
1. הברך שלי נתקעת (אני לא מסוגל להזיז אותה).	1	2	3
2. הברך שלי כואבת בזמן מנוחה.	1	2	3
3. הברך שלי נפוחה.	1	2	3
4. הברך שלי מרגישה חלשה (רועדת).	1	2	3
5. הברך שלי מרגישה לא יציבה (כאילו היא הולכת לקרוס).	1	2	3
6. הברך שלי כואבת כאשר אני מיישר אותה.	1	2	3
7. הברך שלי כואבת כאשר אני עומד הרבה זמן.	1	2	3
8. הברך שלי כואבת בזמן ריצה	1	2	3

LIMB-Q-KIDS™- תסמינים: קרסול וכף רגל

איך מרגישים הקרסול וכף הרגל שלך? אנא השב בהתייחס לשבוע האחרון.

שאלות אלו מתייחסות לרגל שבגללה אתה הולך לרופא.

	תמיד	לפעמים	אף פעם
1. הקרסול שלי כואב בזמן מנוחה.	1	2	3
2. כף הרגל שלי כואבת בזמן מנוחה.	1	2	3
3. הקרסול שלי מרגיש לא יציב (כאילו הוא הולך לְקָרֵס).	1	2	3
4. הקרסול שלי נתקע (אני לא מסוגל להזיז אותו).	1	2	3
5. כף הרגל שלי כואבת בעת הליכה עם נעליים.	1	2	3
6. כף הרגל שלי כואבת בעת הליכה ברגליים יחפות (ללא נעליים על הרגליים).	1	2	3
7. הקרסול שלי כואב כשאני עומד הרבה זמן.	1	2	3
8. הקרסול שלי כואב בעת ריצה.	1	2	3
9. כף הרגל שלי כואבת בעת ריצה.	1	2	3
10. כף הרגל שלי כואבת כשאני עומד זמן רב.	1	2	3

LIMB-Q-KIDS™- תחושת מצוקה הקשורה לרגל

<u>איך אתה מרגיש לגבי הרגל שלך</u>? אנא השב <u>בהתייחס לשבוע האחרון</u>.

שאלות אלו מתייחסות לרגל שבגללה אתה הולך לרופא.

אם אתה משתמש באביזר כמו נעל עם הַגְבָּהָה, מִדְרָס או סד או רגל תותבת, תענה על השאלות תוך מחשבה על הזמן בו אתה משתמש באביזר זה.

	תמיד	לפעמים	אף פעם
1. אני נמנע מלצאת בגלל הרגל שלי (למשל למסיבה).	1	2	3
2. אני מסתיר את הרגל שלי כשאני יוצא.	1	2	3
3. אני מתעצבן כאשר אנשים מסתכלים על הרגל שלי.	1	2	3
4. אני מתעצבן כאשר אנשים שואלים על הרגל שלי.	1	2	3
5. אני נמנע מללבוש מכנסיים קצרים או חצאיות שמראות את הרגל שלי.	1	2	3
6. אני מרגיש לא מרוצה לגבי הרגל שלי.	1	2	3
7. אני לא אוהב איך שאני נראה כשאני הולך.	1	2	3
8. אני לא אוהב איך שהרגל שלי נראית.	1	2	3
9. הרגל שלי גורמת לי להרגיש שונה מאנשים אחרים.	1	2	3
10. אני מודאג לגבי הרגל שלי.	1	2	3
11. אני מתעצבן כאשר הרגל שלי מונעת ממני ליהנות.	1	2	3

LIMB-Q-KIDS™ - תפקוד רגשי

איך אתה מרגיש? אנא השב בהתייחס **לשבוע האחרון.**

	בכלל לא	לפעמים	לעתים קרובות	תמיד
1. אני נהנה מהחיים.	1	2	3	4
2. אני מרגיש מאושר.	1	2	3	4
3. אני מרוצה מהחיים שלי.	1	2	3	4
4. אני אוהב את עצמי.	1	2	3	4
5. אני מרגיש בסדר עם עצמי.	1	2	3	4
6. אני מאמין בעצמי.	1	2	3	4
7. אני גאה בעצמי.	1	2	3	4
8. אני מרגיש בטוח בעצמי.	1	2	3	4
9. אני מרגיש טוב עם איך שאני נראה.	1	2	3	4

LIMB-Q-KIDS™ – תפקוד חברתי

<u>איך החיים החברתיים שלך?</u> אנא השב בהתייחס <u>לשבוע האחרון</u>.

	בכלל לא	לפעמים	לעתים קרובות	תמיד
1. כיף לי עם חבריי.	1	2	3	4
2. אני מקובל על חבריי.	1	2	3	4
3. אנשים מקשיבים למה שיש לי להגיד.	1	2	3	4
4. אנשים מתייחסים אלי כמו לכולם.	1	2	3	4
5. אני אוהב להיות עם אנשים אחרים.	1	2	3	4
6. אני מרגיש שאני משתלב.	1	2	3	4
7. קל לי להכיר חברים.	1	2	3	4
8. אני מרגיש בטוח בעצמי כשאני יוצא (למשל למסיבה).	1	2	3	4
9. חברים מבקשים ממני לצאת איתם.	1	2	3	4
10. אני מרגיש כמו אנשים אחרים בגילי.	1	2	3	4
