# Supplementary figures and images for: Unique Challenges of Hebrew Translation and Cross-Cultural Adaptation of LIMB-Q Kids for Children with Lower Limb Differences
Source: Children (Basel). 2025 Oct 1;12(10):1318. doi: 10.3390/children12101318 (PMC12563977; doi:10.3390/children12101318)

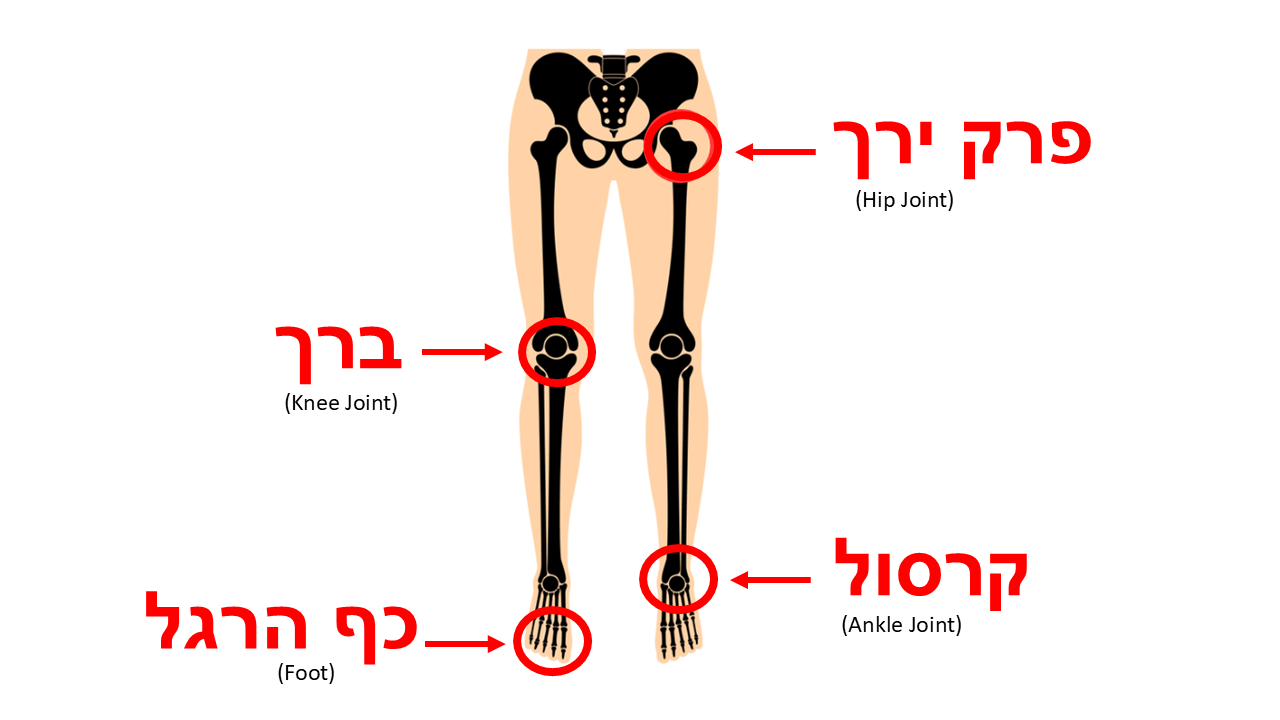

Supplement: Supplementary file 1 [file children-12-01318-s001.zip › Figure S1 Lower limbs with labelled joints and segments.png]
